# Damage of the right dorsal superior longitudinal fascicle by awake surgery for glioma causes persistent visuospatial dysfunction

**DOI:** 10.1038/s41598-017-17461-4

**Published:** 2017-12-07

**Authors:** Riho Nakajima, Masashi Kinoshita, Katsuyoshi Miyashita, Hirokazu Okita, Ryoji Genda, Tetsutaro Yahata, Yutaka Hayashi, Mitsutoshi Nakada

**Affiliations:** 1 Pharmaceutical and Health Sciences, Kanazawa University, Kanazawa, Japan; 2 Department of Neurosurgery, Kanazawa University, Kanazawa, Japan; 3 Department of Physical Medicine and Rehabilitation, Kanazawa University Hospital, Kanazawa, Japan; 4 Department of Neurosurgery, Ishikawa Prefectural Central Hospital, Kanazawa, Japan

## Abstract

Patients with glioma frequently present with neuropsychological deficits preoperatively and/or postoperatively, and these deficits may remain after the chronic phase. However, little is known about postoperative recovery course of right hemispheric function. We therefore studied the characteristics and causes of persistent cognitive dysfunction in right cerebral hemispheric glioma. Eighteen patients who underwent awake surgery participated in this study. All patients who received preoperative neuropsychological examinations were assigned to two groups according to their test results: preoperative deficit and normal. They were reassessed 1 week and 3 months after surgery. The rates of remaining deficits in the deficit group at chronic phase were higher than those of the normal group for all functions. Despite preoperative normal function, the remaining rate for visuospatial cognitive deficits was the highest among all functions. The voxel-based lesion-symptom mapping analysis for visuospatial cognition revealed that a part of the medial superior and middle frontal gyri were resected with high probability in patients with low visuospatial cognitive accuracy. Our study indicates that in patients with preoperative neuropsychological deficits, these deficits tend to remain until the chronic phase. Visuospatial dysfunction frequently persists until the chronic phase, which might reflect damage to the superior longitudinal fasciclus I and II.

## Introduction

Traditionally, since the left cerebral hemisphere has language network, preserving its network during glioma or epilepsy surgery has been considered as essential strategy. However, the right cerebral hemispheric function has been paid less attention, especially the right frontal lobe which has been considered as a silent area. It has gradually been recognized that the right frontal lobe has essential social functions, such as attention, emotion, theory of mind, and visuospatial cognition. Therefore, the preservation of right frontal lobe function has recently been emphasized in glioma surgery^1,2^. In contrast, the usefulness of awake surgery for right parietal glioma to preserve visuospatial cognition has been demonstrated^3^, and some previous reports also supported this idea^4,5^.

Cognitive dysfunctions in patients with glioma are sometimes observed preoperatively because of damage by the tumor itself or by tumor-related factors such as impairments to the cortex or white matter by infiltrating tumor cells, mass effects, edema, and seizure^6,7^. However, preoperative cognitive dysfunction may recover postoperatively. For example, when preoperative cognitive deficits are caused by mass effects or edema, recovery can be expected with high probability^8,9^. By contrast, when deficits are due to white matter damage, only brain plasticity can occur and recovery is not expected^10,11^. Even when preoperative cognitive function is normal, some deficits are occasionally observed following surgery, and we cannot predict whether these deficits are temporary or permanent.

The pattern of postoperative recovery varies depending on the cause of the deficits, the lesion, and the categories of cognitive function. Functional recovery due to the release of mass effects or edema occurs early after surgery, whereas recovery due to brain plasticity needs a relatively long time, ranging over several months^9^. Recent findings have demonstrated that when we attempt to preserve brain function by using awake surgery, cognitive function may decline immediately after surgery, although it usually recovers within 3 months and deficits are less likely to remain until the chronic phase^1,12,13^. Several literatures have demonstrated that some functions are often disturbed after surgery^7,9,14^. For instance, as for patients with gliomas localized in eloquent areas, while recovery of memory function occurred just after surgery and lasted for 1 year, language function was impaired just after surgery and had recovered within 3 months to 1 year^9^. In a study of gliomas localized in the right hemisphere, Yoshii *et al*.^15^ studied several types of cognitive function in 83 patients with glioma at pre- and postoperative 1-month intervals. Their study showed that the cognitive function of patients with low-grade glioma was quite normal preoperatively and was preserved after surgery, while that of patients with high-grade glioma were disturbed preoperatively but recovered after surgery. Although disturbed function may differ depending on the lesion or the resected area, the postoperative course for cognitive function in participants with common lesions has rarely been included in previous studies.

A continuous decline in cognitive dysfunction should be avoided in the chronic phase to preserve the quality of life of patients after surgery. We therefore focused on cognitive dysfunction that persist beyond the chronic phase after surgery. We hypothesized that functional recovery following surgery may relate to preoperative neuropsychological function. The aim of this study was to examine the characteristics and causes of persistent cognitive dysfunction in patients with right cerebral hemispheric lower grade glioma.

## Results

### Clinical and Neuropsychological data

**Figure 1:**
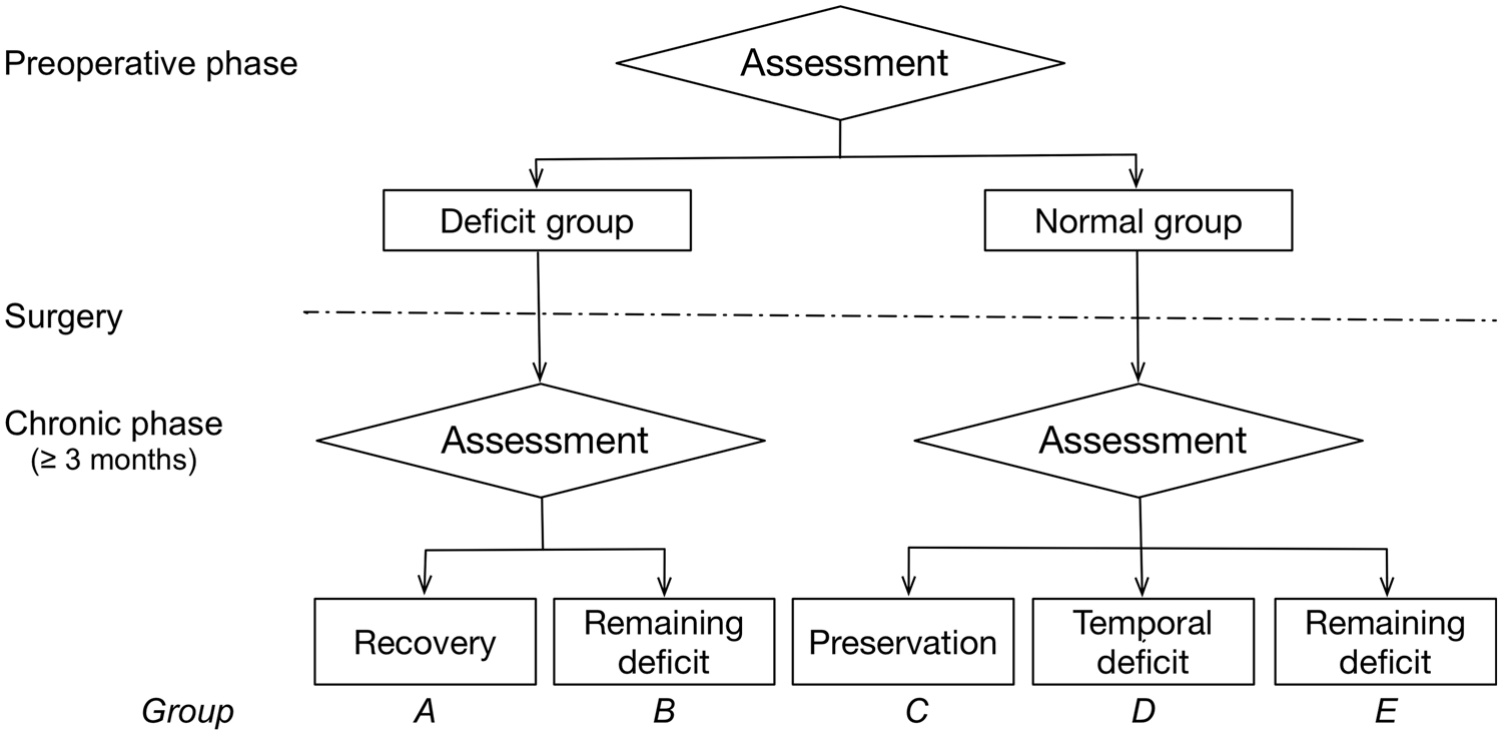
Five groups which were formed depending on the deficit and clinical course. Patients were assigned to the five groups according to each function (processing speed, fluency, spatial working memory, emotion, ToM and social cognition, visuospatial cognition). Groups A and B: deficits were observed preoperatively; A, deficit recovered within 3 months; B, deficit remaining until the chronic phase. Groups C, D, and E: no deficits were observed during the preoperative phase; C, normal at the chronic phase; D, deficits occurred just after surgery, but recovered by the chronic phase; E, deficits occurred after surgery and had not recovered within 3 months.

**Figure 2:**
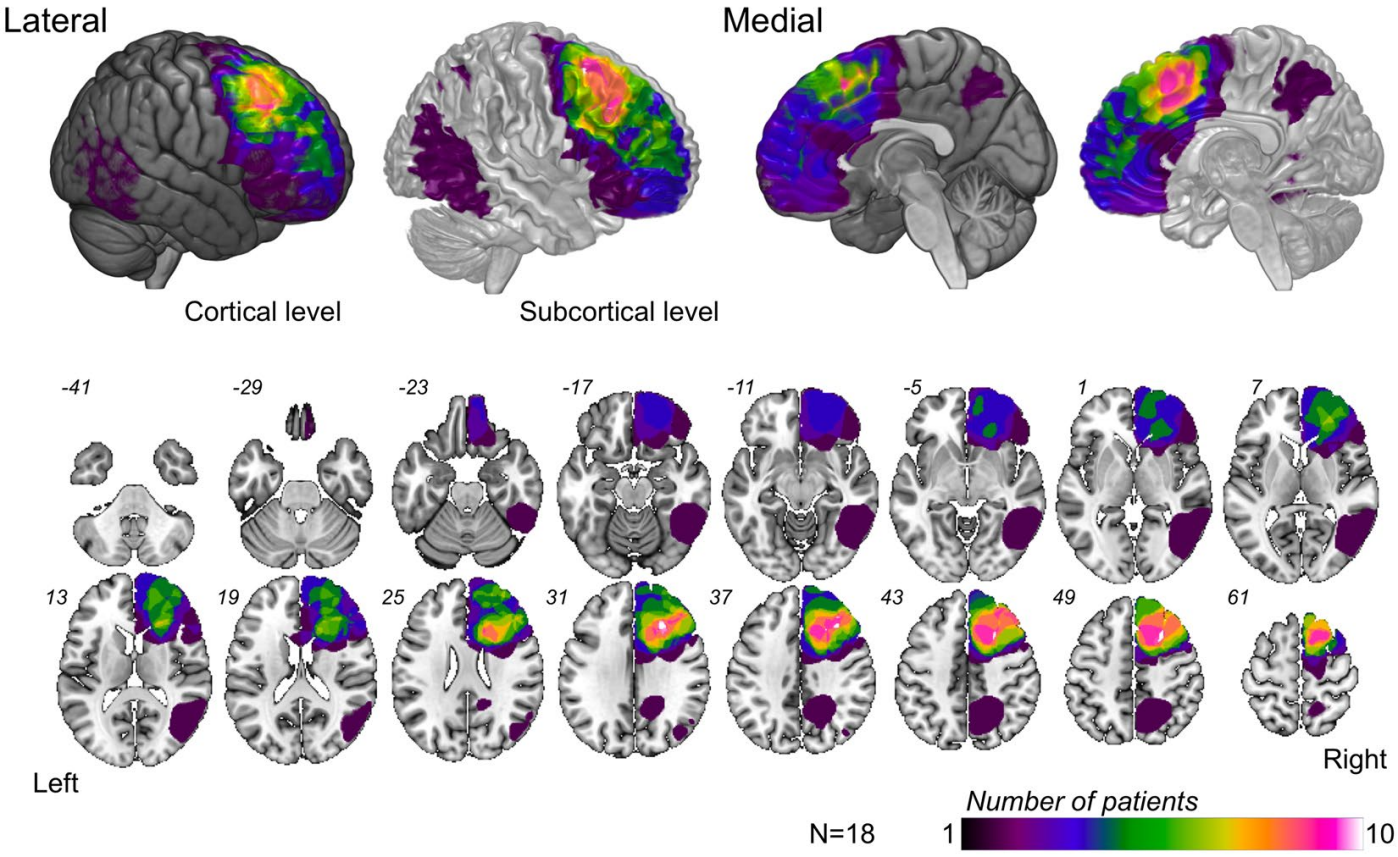
Overlap map of the resection cavity. Overlap map of resection cavities shows that the right superior frontal gyrus was the region with the greatest overlap (n = 10). As for subcortical level, the greatest overlap region was equivalent to the course of the cingulum and SLFs I and II.

Several neuropsychological assessments were performed before surgery, and patients were divided into two groups; namely, the normal and preoperative deficit groups according to the results of neuropsychological assessments for every function (Fig. 1). Following that, they classified it into five subgroups (A to E) according to their postoperative course. Figure 2 shows the overlap map of resection cavities of all patients (n = 18). The right superior frontal gyrus was the greatest overlap of resection cavity in cortical level (n = 10). In subcortical level, the greatest overlap of the region was equivalent to the course of the cingulum and superior longitudinal fascicles (SLF) I and II.

**Figure 3:**
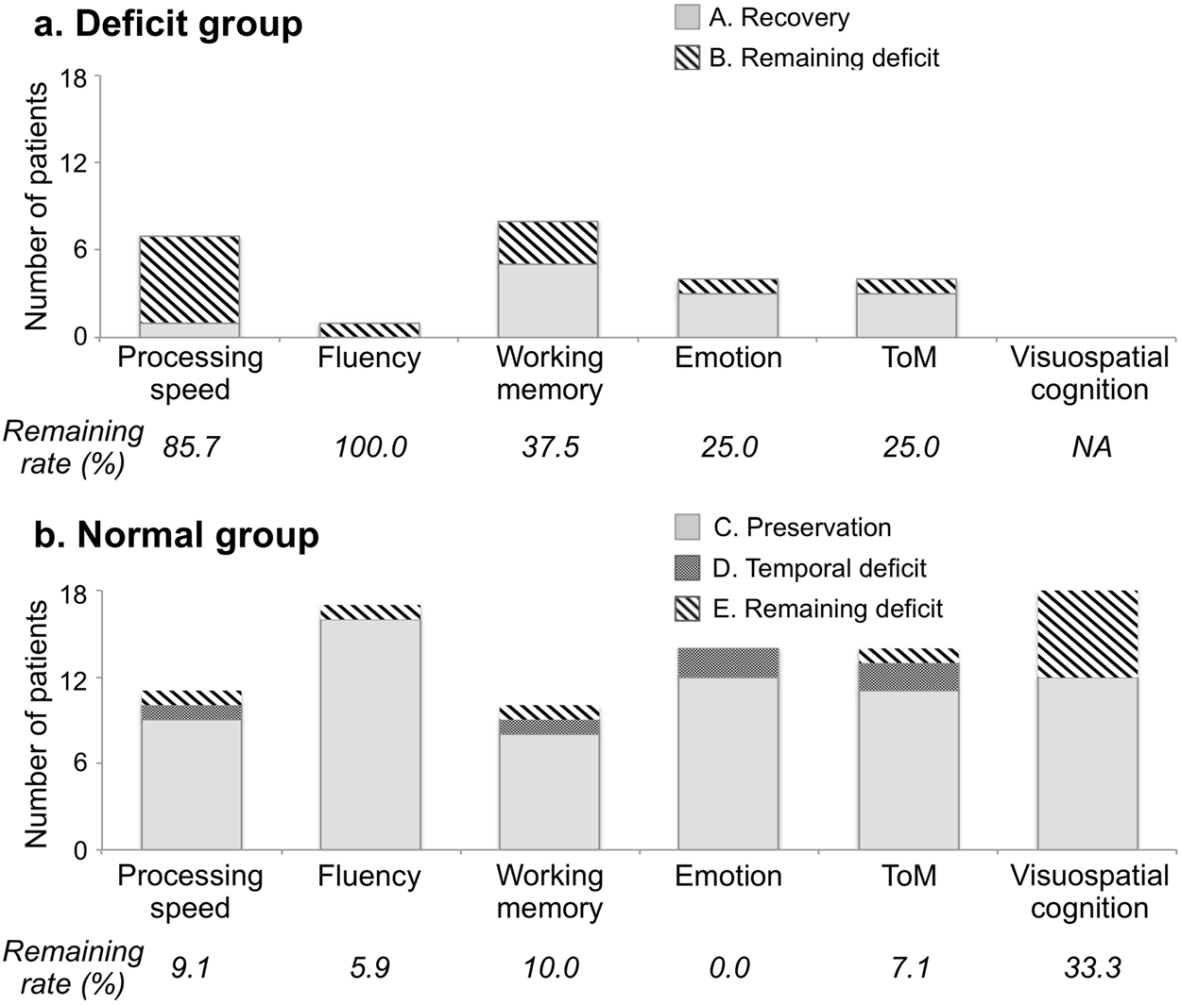
Symptoms at the chronic phase. Remaining rates (%) of preoperative deficit group (**a**) were worse than preoperative normal group (**b**) in all functions. ToM, theory of mind/social cognition; Working memory, spatial working memory; Remaining rate, ratio of patients with remaining deficit to all patients from each group at 6 months defined by B/(A + B) and E/(C + D + E).

The results of neuropsychological assessments at the chronic phase are shown in Fig. 3 (see also Supplementary Table 1). The ratio of the numbers of participants with remaining deficits, namely the remaining rates B/(A + B) and E/(C + D + E) were calculated for each group. As shown in Fig. 3a, the mean score for the remaining rate in the preoperative deficit group (Groups A and B, 50.0%) was larger than that of the preoperative normal group (Groups C, D, and E, 11.9%). In the preoperative normal group, processing speed, fluency, working memory, emotion, and Theory of mind (ToM)/social cognition had recovered by 3 months, even if some patients had declined to a subnormal level just after the surgery. By contrast, deficits in visuospatial cognition remained in 33.3% of patients in the preoperative normal group. Although the visuospatial cognitive assessment showed no deficit before surgery, the remaining rate in Group E was higher (33.3%) than any other functions (Fig. 3b).

**Figure 4:**
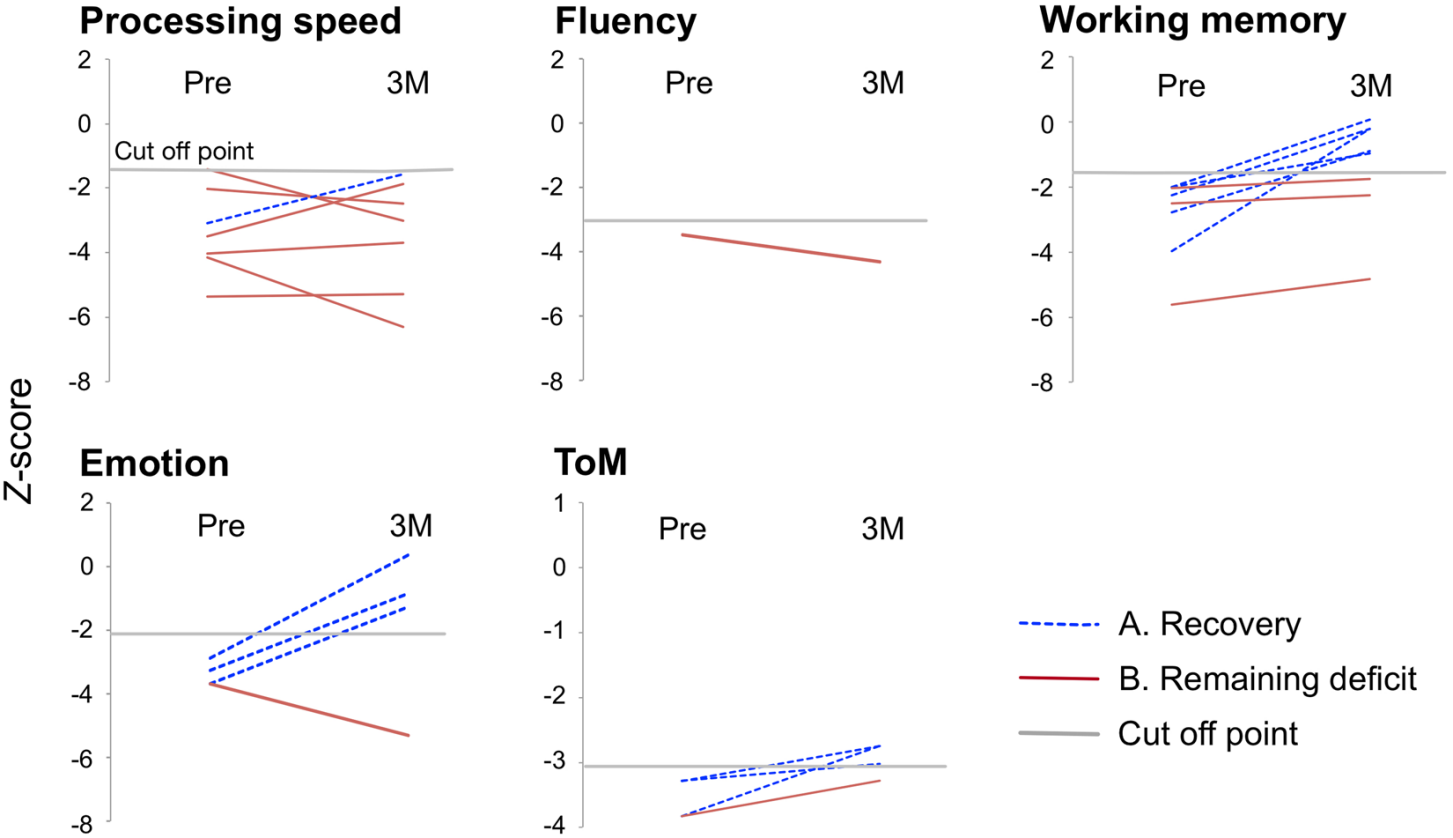
Progress in neuropsychological functioning by the preoperative deficit group. Grey line indicates the cut-off point of each test described in the Supplementary Table 2. Even though their cognitive functions were disturbed preoperatively, cognitive functions of 80% patients were preserved preoperative level or recovered.

In the deficit group, the process of recovery was shown (Fig. 4). Comparing preoperative *Z*-scores for deficit group with postoperative scores at 3 months, 80% of patients achieved preservation or recovery. On the other hand, in patients whose score decline at chronic phase, their Z-score were declined slightly (<1.0 points) in 2 patients (8.3%), and declined moderately (1.6 to 2.1 points) in 3 patients (12.5%).

Case 7 was transferred to another affiliated hospital to continue rehabilitation since his transient hemiparesis had worsened because of Supplementary motor area (SMA) syndrome, but he returned to his previous employment at 5 months after surgery. Case 11 could not resume his previous social life due to a decline in motivation and social behavioural problems after discharge. Within 3 to 6 months of surgery, all patients except for Case 11 had continued their previous social lives.

### Visuospatial cognition

**Figure 5:**
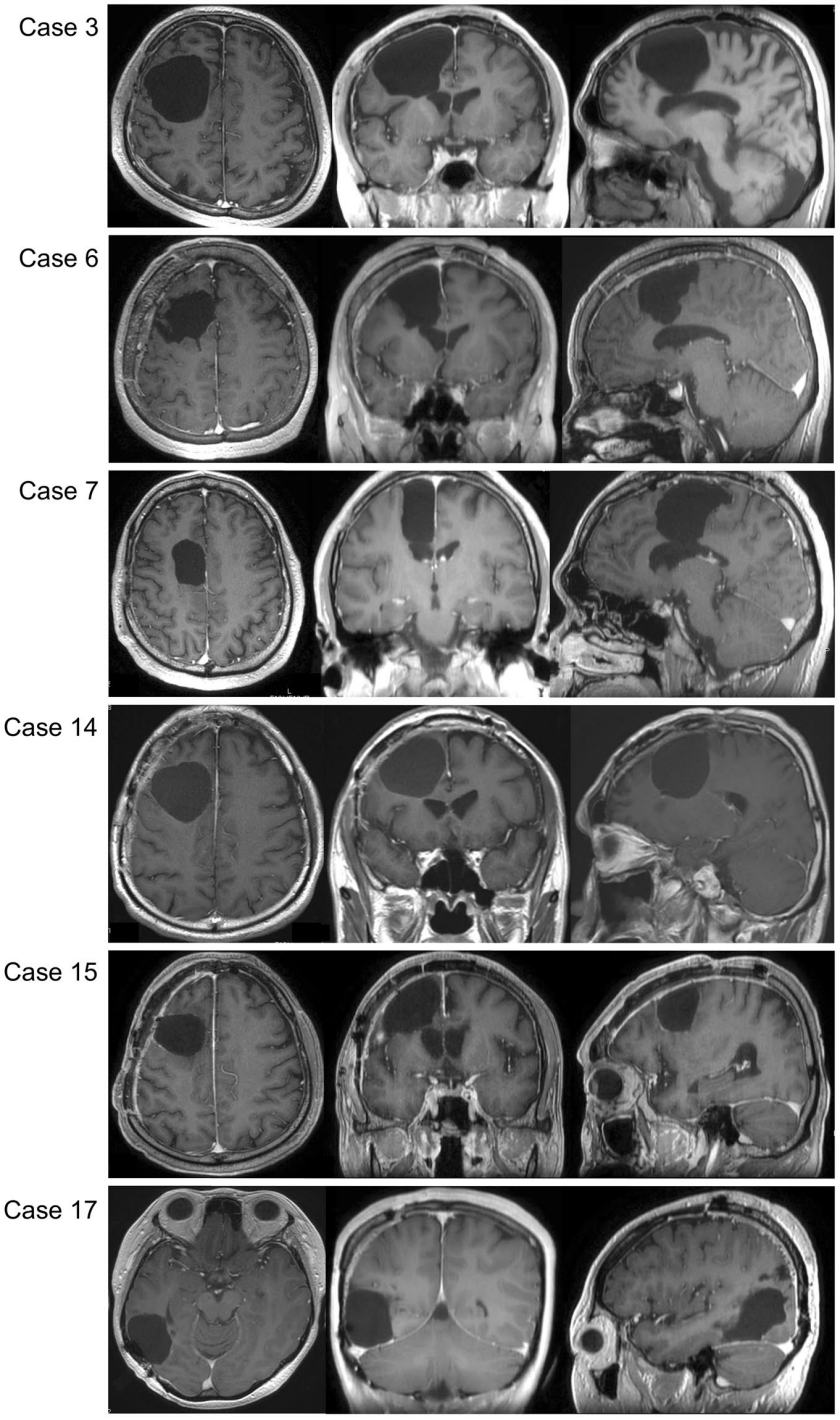
Postoperative MR images of patients whose visuospatial cognitive lasted ≥3 months. In the left, middle and right columns, T1 weighted MR images of axial, coronal, and sagittal slices, respectively, are shown for each patient. Resection cavities are in the middle frontal gyrus and/or superior frontal gyrus including the medial region in patients with frontal lesion.

**Figure 6:**
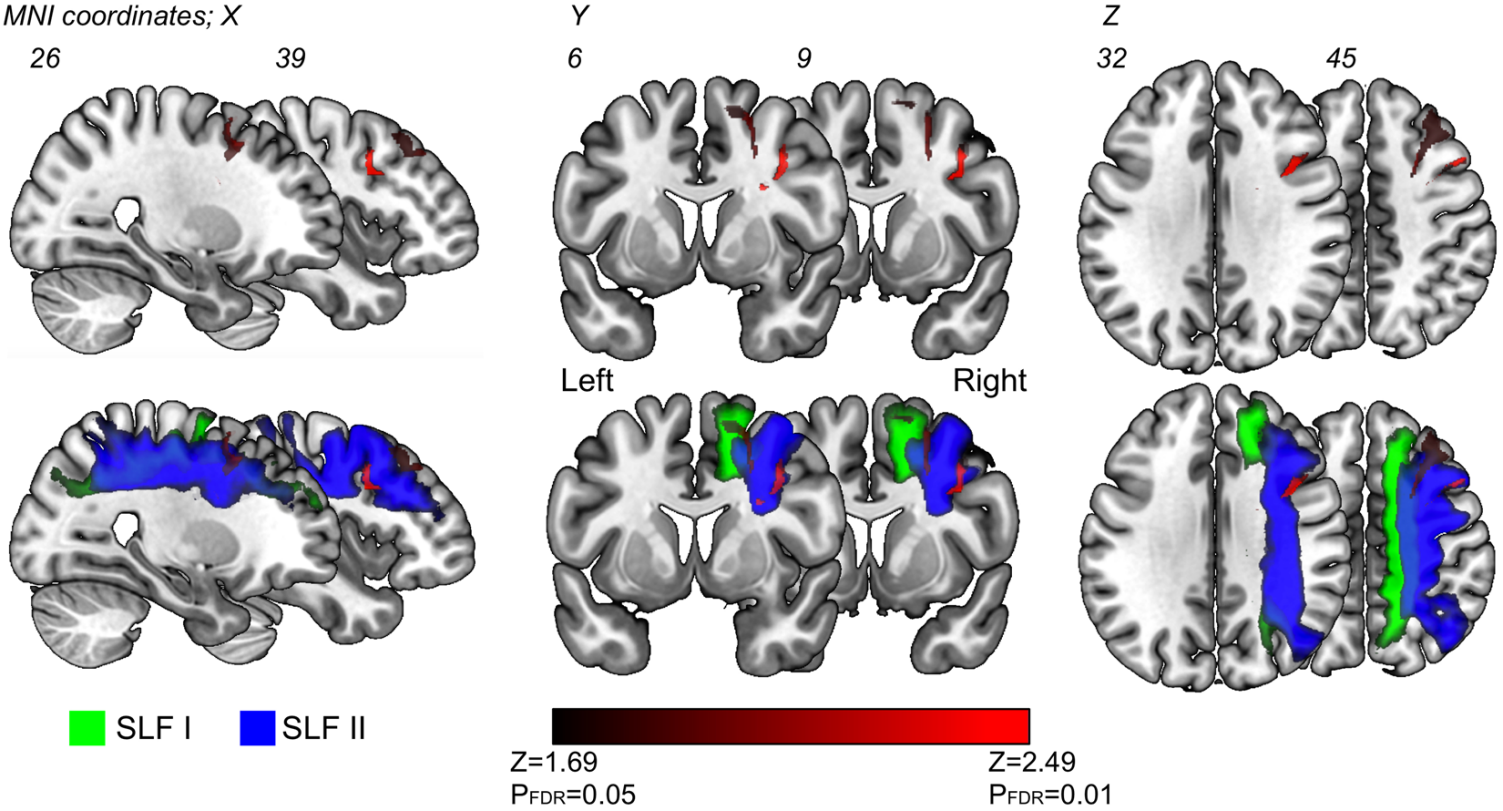
The result of the VLSM analysis. Statistically significant clusters for the resection cavity identified using the line bisection test scores (upper column) were overlapped with the SLF I (green) and SLF II (blue) (lower column). The statistical map shows only significant voxels with a FDR-controlled threshold (*p* = 0.05; *z* = 1.69).

**Figure 7:**
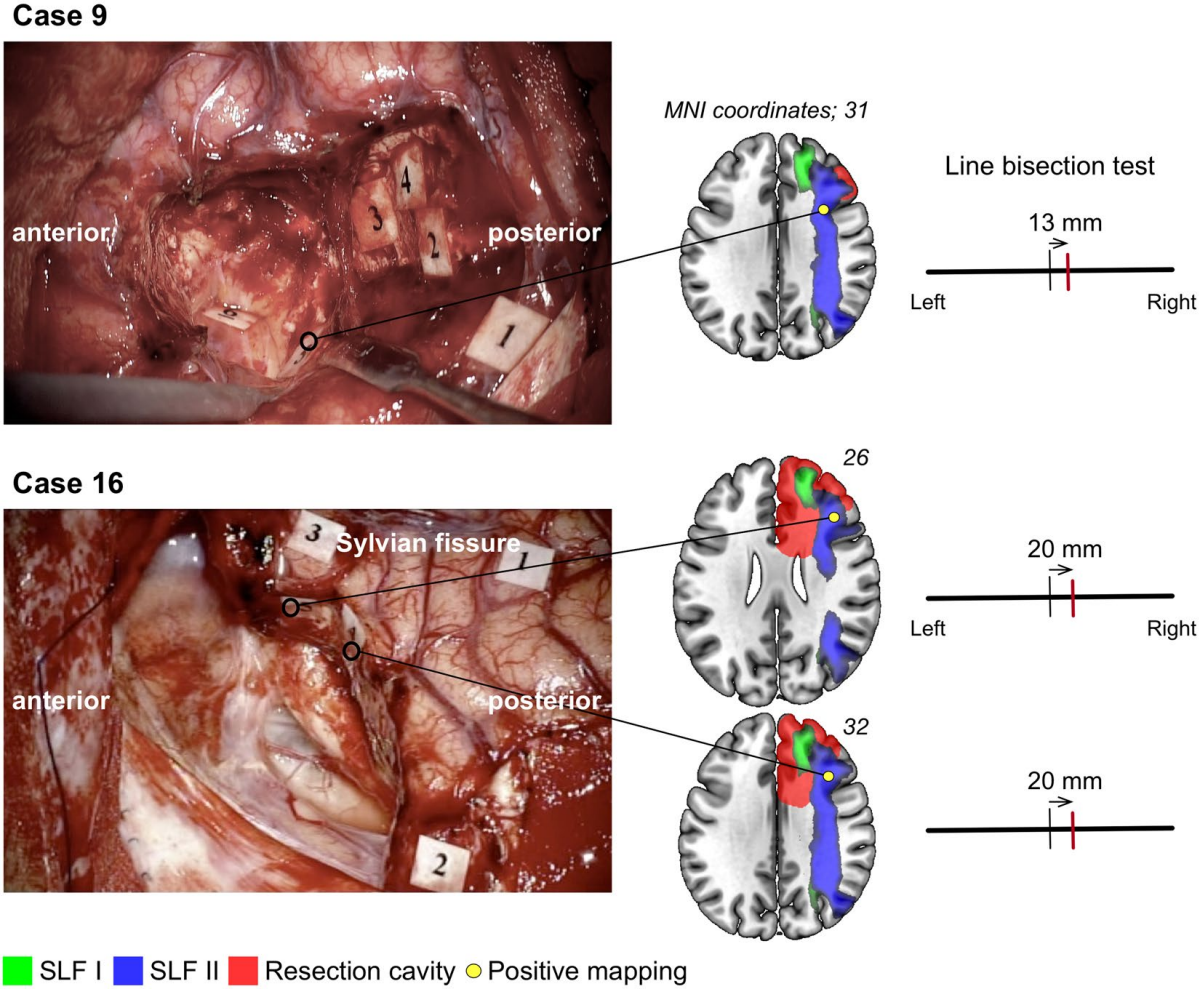
Illustrative cases (Case 9 and 16) of intraoperative visuospatial cognitive assessment. Intraoperative photograph shows positive mapping site during intraoperative visuospatial cognitive assessment (Tag 5 in Case 9, and Tag 4 and 5 in Case 16). The stimulation of the subcortical region elicited rightward deviation (13 mm in Case 9 and 20 mm in Case 16) in the line bisection test. To demonstrate the spatial relationship among the SLFs I (green) and II (blue), resection cavities (red), and positive mapping sites (yellow circles), all these structures are displayed together. Case 9: Tag 1, 2, 4, Anarthria; Tag 3, initiation disorder of speech; Tag 5, error in line bisection test; Tag 6, error in spatial working memory. Case 16; Tag 1, movement disorder; Tag 2, 3, error in theory of mind.

To investigate surgical influences, we have focused visuospatial cognition in current our research, since the postoperative remaining rate was the highest despite normal preoperative function. In patients with remaining deficits, magnetic resonance (MR) images showed resection of the middle frontal gyrus and/or the dorsal and medial superior frontal gyrus, for five out of six patients (Fig. 5). The voxel-based lesion-symptom (VLSM) analysis was performed to evaluate the anatomical correlates of visuospatial cognitive accuracies and specific association areas. We used as a variable the average of the absolute value of the amount of deviation. Figure 6 shows the statistical map from the VLSM analysis performed on the line bisection test. The largest cluster of significant voxels was located in the deep region of the medial superior frontal gyrus and middle frontal gyrus (*Z* max = 2.83; *p* < 0.05, FDR corrected). Following this, the VOI of significant voxels was overlaid with the VOIs for the SLFs I and II on the MNI template (Fig. 6, lower column). The result showed that the statistically significant clusters from the VLSM analysis (*p* < 0.05) were located on the middle of the SLFs I and II and the external side of the SLF II (the VLSM analyses for other functions, see Supplementary Figure 1). In postoperative neuropsychological examination, degree of abnormal deviations of line bisection test at postoperative 3 months were 7–13 mm left-ward deviation in 5 cases (Cases 6, 7, 14, 15 and 17) and 7–14 mm right-ward deviation in 1 case (Case 3). Unfortunately, these 6 patients complained related to visuospatial cognitive deficit in their social lives, even after they returned to their work. Though these patients could easily perform self care activities, their deficit influenced on the activity which needed high attention, such as driving or office job. Their deficit lasted more than 1 year after surgery. Importantly, as for these patients with frontal lesion, they could not perform the line bisection test during awake surgery, because of movement disorders of upper limb induced by intraoperative SMA syndrome^16^. Consequently, we failed to assess visuospatial cognition accurately in 5 patients with frontal lesion. In contrast, in patients whose visuospatial cognition could be assessed and preserved during awake surgery, their postoperative visuospatial cognition was successfully preserved, except Case 17. Two illustrative cases are shown in Fig. 7. The positive mapping site of subcortical region is located on the course of the SLF II. Additionally, in patients who showed deficit and recovery, deep parts of the superior frontal gyrus and middle frontal gyrus were preserved except in Cases 5 and 16 (Supplementary Figure 2).

## Discussion

To investigate the characteristics and courses of the neuropsychological deficits that persist beyond the chronic phase in patients with right cerebral hemispheric glioma, we divided the patients into normal and deficit groups based on the results of preoperative neuropsychological assessments, and examined changes in their cognitive function. We found that preoperative neuropsychological deficits are liable to remain with high probability beyond the chronic phase, in contrast with the outcomes observed in patients who have normal preoperative cognitive function. This data suggests the advantage of early operation before appearance of neuropsychological deficits for WHO grade II and III glioma to preserve the cognitive functions.

Recently, though growing number of studied have emphasize on the importance of awake surgery for preserving brain functions^12,13,17^, no studies have focused on awake surgery for patients who suffered any cognitive dysfunctions before surgery. In this study, we focused on those kinds of patients, and we demonstrated that performing surgery before the onset of a deficit and preserving white matter with a primary function led to better neuropsychological outcomes. Notably, 80% of patients with decreased neuropsychological function at the preoperative phase in our study, showed functional recovery or preservation by 3 months postoperatively. Awake surgery enhances the ability to avoid any remaining deficit derived from damage to the white matter^5,18,19^. Moreover, even if transient deficits are observed following surgery, we might be able to anticipate further recovery due to brain plasticity^1,12,13,20^.

In our data, visuospatial cognition was rarely disturbed at the preoperative phase in patients with WHO grade II and III glioma. However, once a deficit occurs following surgery, it will remain with a high probability of as much as 33%. Moreover, our analyses demonstrated that deficits in visuospatial cognition persisting to the chronic phase may reflect damage to the dorsal SLF, namely the SLFs I and II. The results of line bisection test showed the deficit of visuospatial cognition in our cases is not unilateral spatial neglect as recognized by the right parietal lobe injury^3^. We speculate that the deficits of visuospatial cognition in our cases are attributed to the disorders of the attention system. Previous studies showed that the attention system consists of two networks, the dorsal network and the ventral network^21–24^. The dorsal network overlaps with the SLF I, which connects the superior parietal and superior frontal regions and is involved in top-down attention^23,24^. Top-down attention operates when attention is focused voluntarily on visual stimuli. The ventral network overlapping with the SLF III is involved in bottom-up attention, namely stimulus-driven attention, which works involuntarily when sensory stimulation is received^23^. The SLF II overlaps with both the ventral and the dorsal networks and communicates directly with the SLFs I and III^25^. Considering both networks can flexibly modulate their interactions to control attentional networks successfully, damage to either the SLF I or II might result in a remaining deficit in visuospatial cognition as a part of a derangement of the attentional networks. Previous studies have revealed that damage to the SLF II by stroke or brain tumor induces deficits in visuospatial cognition^5,26,27^. In line with previous studies, our study also suggests that damage to the SLF II by surgery has a major impact on long-lasting visuospatial cognitive deficits. We also found that the region corresponding to the SLF I was also resected with relatively high probability in patients with visuospatial cognitive deficits. However, damage solely to the SLF I is less likely to result in a permanent deficit in visuospatial cognition^21,22^. Further analysis with additional cases will be necessary to investigate the unanswered question whether the sole damage of the SLF I can induce the deficits of visuospatial cognition.

The explanation for the inability of preservation of visuospatial cognition in patients with frontal lesion by awake surgery may lie in the following reason. Surgery to the medial superior frontal region is liable to cause the movement disorder implicated in intraoperative SMA syndrome^16^. Under such circumstances, the line bisection task is difficult to perform as an intraoperative assessment. In fact, in 5 patients whose deficit remained until the chronic phase, we could not use the line bisection test because a disturbance to the upper limb appeared during the last half of surgery due to intraoperative SMA syndrome. Since damage to these regions frequently leads to persistent visuospatial dysfunction, further studies are required regarding the intraoperative assessment of visuospatial cognition in patients with glioma that is localized to the medial superior frontal gyrus.

There are some limitations in this study. First, despite brain mapping, it is sometimes difficult to completely avoid post-operative deficit, especially disorders of visuospatial cognition. When we use intraoperative assessment, it should be kept in mind that during these tasks, which require high-level function, correct judgment may be influenced by multiple factors as patient’s concentration, motivation, and fatigue. Second, from the statistical standpoint, statistical power of this study is low because of limited number of participants. However, some studies reported successful results using neuro-imaging analysis with small number of patients as our study^28,29^. Further study with large number of patients will be required to demonstrate our results with strong evidence. Third, participants in this study included those with WHO grade II and III. Clinically, cognitive deficits in patients with grade II or III rarely occurred preoperatively because of the low impact of mass effects and the reorganization that occurs in reaction to the slow growth of the tumor^30^. However, since concordant results have not been obtained regarding relationship between neuropsychological findings and WHO grades^15,31^, further studies are required. Fourth, unexpectedly, resected volume of cortical and subcortical region not always corresponds to severity of neuropsychological dysfunction (See also Fig. 5 and Supplementary Figure 2). Further study will be required for the inexplicable result. Finally, the existence of the SLF I of human is matter of debate. From the past, the existence of the SLF I has been demonstrated using neuroimaging, including autoradiography, diffusion tensor imaging, diffusion spectrum imaging, and spherical deconvolution^32–34^. However, in fiber dissection study, there are conflicting opinions as for the existence of the SLF I. Most recently, only one fiber dissection research provided by Yagmurlu^35^ successfully identified the SLF I as an isolated long association fiber. On the contrary, Wang^36^ indicated that the SLF I could not be found as an isolated fiber, instead as a component of the cingulum. We have to keep this issue in mind, although discussion the existence of the SLF I goes beyond the scope of this study.

## Conclusions

When neuropsychological deficits are observed preoperatively in the patients with right cerebral hemispheric low and intermediate grade glioma, these deficits are more likely to remain until the chronic phase compared with those in the patients with normal preoperative functioning. Additionally, visuospatial cognitive disturbances following surgery can be caused by damage to the dorsal SLF, and cannot be reversed.

## Methods

### Patients

Eighteen patients with World Health Organization (WHO) grade II or III glioma localized in the right frontal and parietal lobe participated in this study. Their average age was 46.6 ± 11.1 years (mean ± *SD*). The patients underwent awake surgery in Kanazawa University Hospital between September 2013 to March 2017 and were followed up for more than 3 months after surgery. Patient details are shown in Table 1. Written informed consent was obtained from all individual participants included in the study. This study was performed according to the guidelines of the Internal Review Board of Kanazawa University, and was approved by the medical ethics committee at Kanazawa University (No. 1797).

**Table 1:** Patient characteristics.

Case	Age, Sex	Lesion	Diagnosis	Symptoms	Removal	RT, CTx
1	57, M	frontal	diffuse astrocytoma	incidental	PR	no
2	41, F	frontal	diffuse astrocytoma	incidental	GTR	no
3	34, M	frontal	diffuse astrocytoma	headache	GTR	no
4	31, F	frontal	diffuse astrocytoma	seizure	STR	no
5	39, M	frontal	diffuse astrocytoma	seizure	GTR	no
6	63, F	frontal	oligodendroglioma	headache	GTR	no
7	38, M	frontal	oligodendroglioma	seizure	PR	no
8	37, F	frontal	oligodendroglioma	seizure	GTR	no
9	40, F	frontal	oligodendroglioma	seizure	GTR	no
10	37, F	frontal	anaplastic astrocytoma	headache	GTR	RT + CTx
11	68, M	frontal	anaplastic astrocytoma	seizure	PR	RT + CTx
12	43, F	frontal	anaplastic oligodendroglioma	seizure	STR	CTx
13	47, F	frontal	anaplastic oligodendroglioma	headache	STR	CTx
14	61, M	frontal	anaplastic oligodendroglioma	seizure	PR	CTx
15	64, M	frontal	anaplastic oligodendroglioma	incidental	STR	CTx
16	40, F	frontal	anaplastic oligodendroglioma	seizure	GTR	RT + CTx
17	48, F	parietal	anaplastic oligodendroglioma	headache	PR	CTx
18	51, M	parietal	anaplastic oligodendroglioma	sensory impairment	PR	CTx

M, male; F, female; GTR, gross total resection; STR, subtotal resection; PR, partial removal; RT, radiotherapy; CTx, chemotherapy.

### Neuropsychological assessment

Patients were divided into two groups; namely, the normal and deficit groups according to the results of preoperative neuropsychological assessments for every function (Fig. 1). The two groups were divided based on a cut-off score or a threshold of >mean − 2*SD* for normal Japanese (Supplementary Table 2). Since cut-off scores for the line bisection test varied depending on reports, we used the standard described by Azouvi^37^ that has been often used in awake surgery. These neuropsychological assessments were collected in the noise controlled room by the same trained occupational therapist (R.N.). We again investigated the same types of neuropsychological assessment for both groups at 3 months and grouped them further into Groups A to E. In the deficit group, two groups were defined as “deficit recovered within 3 months” (Group A) or “deficit remained until the chronic phase” (Group B). In the normal group, three groups were defined as “normal at the chronic phase” (Group C), “deficits occurred just after surgery with recovery by the chronic phase” (Group D), and “deficits occurred after surgery without recovery within 3 months” (Group E) (Fig. 1). As for group C and D, though all patients recovered until chronic phase, we divided them into two groups. Some previous reports provide evidence that even if some functions in dominant cerebral hemisphere are disturbed just after surgery, they recover within 3 months with high possibility^12,13^. Nonetheless, clinically, not all functions follow same course postoperatively. That is the reason why we chose this grouping, and that will give us better understanding about characteristics and causes of cognitive dysfunction after surgery. All raw test scores were transformed into age-adjusted *Z*-scores based on average values of healthy subjects, and age-related *Z*-scores obtained before and 3 months after surgery in the deficit group were compared.

The following neuropsychological assessments were used: 1) letter cancellation test (time required)^38^ for processing speed, 2) verbal fluency test^39^ for fluency, 3) spatial 2-back test^40^ for spatial working memory, 4) expression recognition test for adults^41^ for emotion, 5) picture arrangement task of Wechsler adult intelligence scale - third edition^42^ for ToM and social cognition, 6) line bisection test^43^ for visuospatial cognition.

### Other measures

All patients were operated on by using an asleep-awake-asleep technique with cortical and subcortical brain mapping achieved by electrical stimulation^44^. Several tasks were performed intraoperatively, and were selected according to preoperative function, motivation of patients, social background, and needs of patients. The tasks included a dual task in which patients perform two tasks simultaneously such as picture naming and movement of an upper extremity, Stroop test, spatial 2-back test, expression recognition test, ToM test, line bisection test. To note, we have selected appropriate intraoperative tasks for each patient from above mentioned tasks based on the optimal onco-functional balance in each patient. However, Cases 3, 6, 7, 14, and 15 could not perform line bisection test during the last half of awake surgery, because of movement disorders induced by intraoperative SMA syndrome^16^.

### MR images and lesion mapping

Structural MR images were acquired at the time of the behavioural assessment as a part of their standard care. The images had been acquired using conventional high-resolution 3DT1-weighted sequences on a 3.0 Tesla MRI scanner (Signa Excite HDx 3.0T, General Electric Medical Systems). MR images were normalized to the Montreal Neurological Institute (MNI) template using SPM12 (http://www.fil.ion.ucl.ac.uk/spm/software/spm12/) implemented in the Matlab environment (http://www.mathworks.com/products/matlab/) and then resection cavities in all patients were reconstructed using MRIcron software (http://www.mccauslandcenter.sc.edu/mricro/mricron/). Each reconstruction was first achieved by R.N. and inspected by a neurosurgeon (M.K.).

### Voxel-based lesion-symptom mapping analysis

To demonstrate the putative relationship between the tests scores and the location of the resection cavity, the VLSM analysis was performed as previously described using NPM software provided in the MRIcron package^29^. The dependent variables were tests’ score of each neuropsychological examination. For instance, as for line bisection test, degree of deviation (mm) from the mid-points were used for dependent variables (See also Supplementary Table 2). Only voxels that showed damage in >15% of the subjects were included as previously reported^45,46^. The parametric *t*-test was chosen to generate the statistical maps. A FDR correration was systematically applied to control false positive error, with a threshold of *p* = 0.05. The significant differences between with and without lesion were identified and presented as Z scores at MNI coordinates. To investigate the potential roles of the SLF, a standardized white matter atlas was used^47^. The VOI of the VLSM analysis was overlapped with the regions encompassing more than 50% of SLF I (green) and SLF II (blue) on the MNI template.

## Electronic supplementary material

Supplementary Information
