# Supplementary material for: Damage of the right dorsal superior longitudinal fascicle by awake surgery for glioma causes persistent visuospatial dysfunction
Source: Sci Rep. 2017 Dec 7;7:17158. doi: 10.1038/s41598-017-17461-4 (PMC5719443; doi:10.1038/s41598-017-17461-4)
Supplement: Supplementary file 1 — Supplementary Information [file 41598_2017_17461_MOESM1_ESM.pdf]

Supplementary Information

Damage of the right dorsal superior longitudinal fascicle by awake surgery for glioma causes persistent visuospatial dysfunction

Riho Nakajima,[1] Masashi Kinoshita,[2] Katsuyoshi Miyashita,[2] Hirokazu Okita,[3] Ryoji Genda,[3] Tetsutaro Yahata,[3] Yutaka Hayashi,[4] Mitsutoshi Nakada[2*]

[1]Pharmaceutical and Health Sciences, Kanazawa University, Kanazawa, Japan

[2]Department of Neurosurgery, Kanazawa University, Kanazawa, Japan

[3]Department of Physical Medicine and Rehabilitation, Kanazawa University Hospital, Kanazawa, Japan

[4]Department of Neurosurgery, Ishikawa Prefectural Central Hospital, Kanazawa, Japan

* **Corresponding author:** Mitsutoshi Nakada, M.D.

Supplementary table 1. Neuropsychological function at chronic phase

Case	Processing speed	Fluency	Spatial working memory	Emotion	ToM/ social cognition	Visuo-spatial cognition
1	E	C	A	A	D	C
2	D	C	C	C	C	C
3	B	C	A	C	C	E
4	C	C	C	C	C	C
5	B	C	C	A	C	C
6	C	C	D	D	A	E
7	C	C	C	C	C	E
8	C	C	C	C	C	C
9	C	C	C	C	C	C
10	C	C	E	C	C	C
11	B	B	B	B	A	C
12	C	C	A	D	D	C
13	C	C	A	C	A	C
14	B	E	A	A	B	E
15	B	C	C	C	E	E
16	C	C	B	C	C	C
17	B	C	C	C	C	E
18	A	C	B	C	C	C

A and B; Preoperative deficit group; A, recovery; B, remaining deficit. C, D, and E; preoperative normal group; C, preservation; D, temporal deficit; E, remaining deficit (see also Fig. 1).

Supplementary table 2. Cut off score of each test

Functions	Tests	Unit	Cut off point
Processing speed	Letter cancellation test	(sec)	cut off score of each age
Fluency	Verbal fluency test	(words)	< 10 words
Spatial working memory	Spatial 2-back test	(%correct)	< 69.4% *1
Emotion	Expression recognition test for adults	(score)	< 15 *2
Theory of mind/ Social cognition	WAIS-III, picture arrangement task	(age-adjusted scale score)	< 5
Visuospatial cognition	Line bisection test	(mm)	< 6.5mm, more than 2 of 3 lines

Spatial 2-back test and Expression recognition test are based on our own data; Participants, right handed healthy volunteers (n=18), aged 45.9 +/- 8.4 years old; *1 mean +/- SD = 91.3 +/- 10.4, cut off score ≤ mean – 2SD (69.4); *2 mean +/- SD = 20.1 +/- 2.5, cut off score ≤ mean – 2SD (15.2). WAIS, Wechsler adult intelligence scale - third edition.

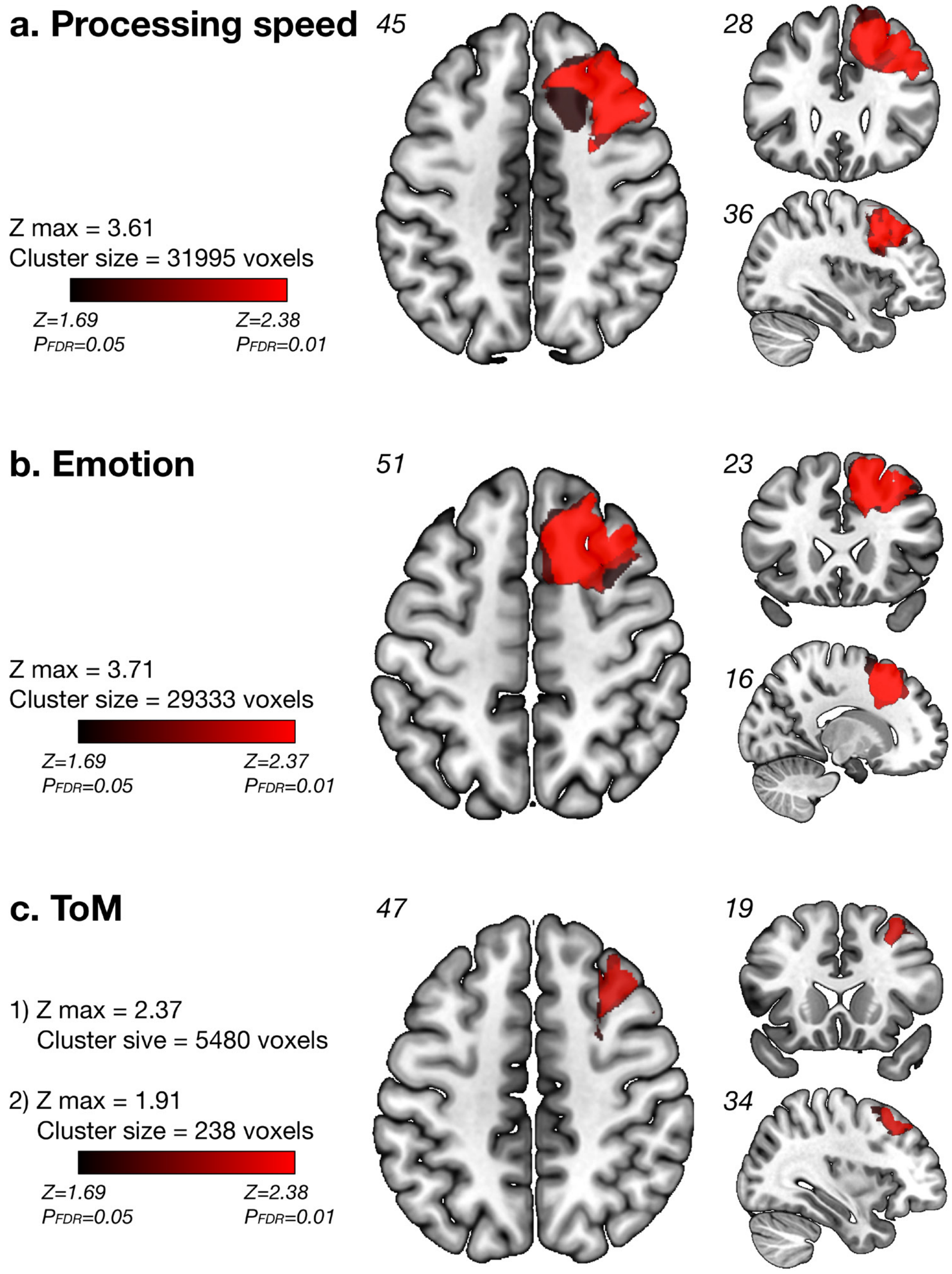

Supplementary Fig S1. Results of VLSM analyses for all functions

At postoperative 3 months, statistically significant regions were found in processing speed (a), emotion (b), and Theory of mind (ToM)/social cognition (c). We could not found significant regions for fluency and working memory at chronic phase. To note, the statistical procedure was exactly same as described in the main text.

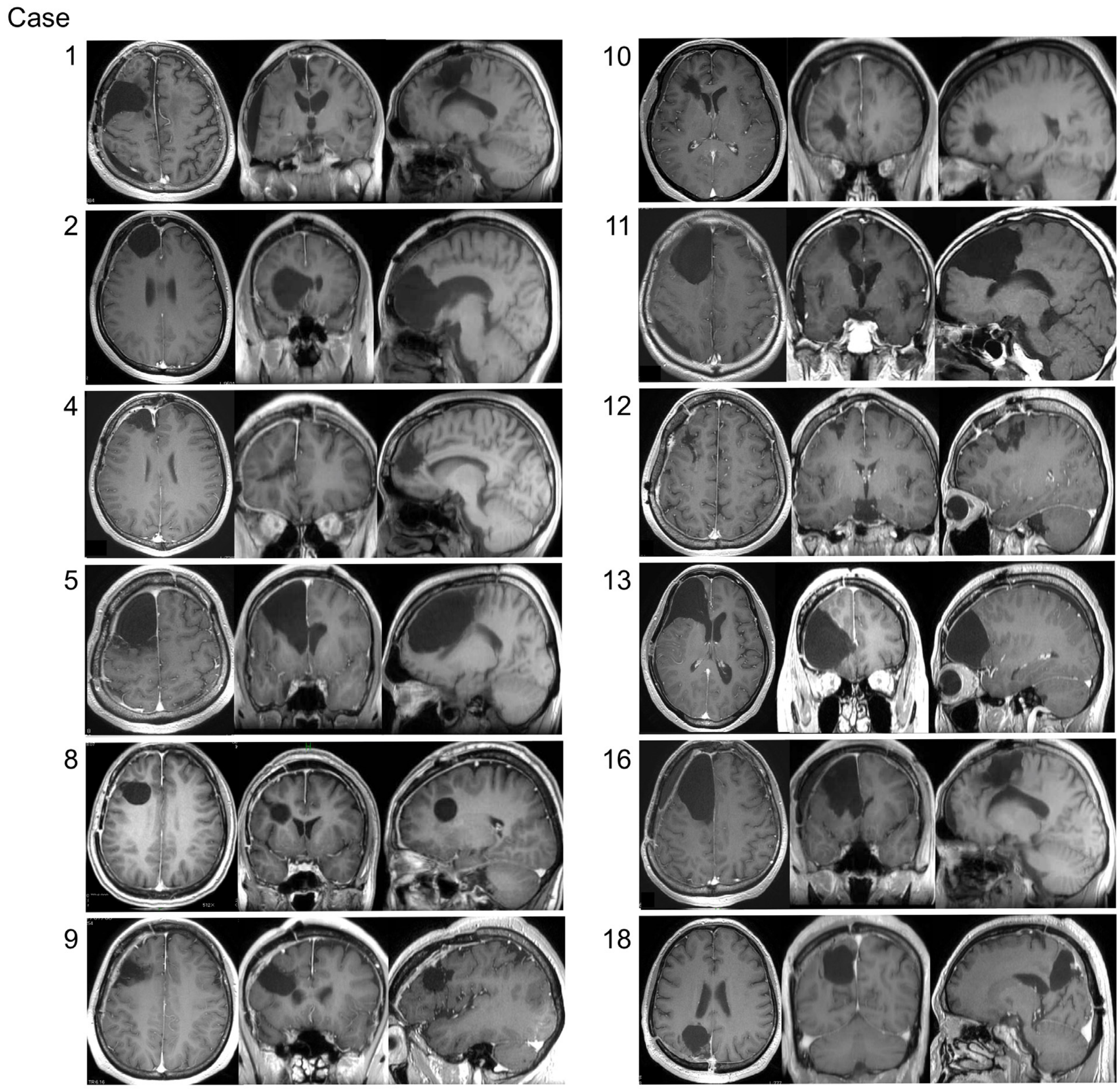

Supplementary Figure S2. Postoperative MR images of patients whose visuospatial cognition was normal at 3 months.

The left, middle and right columns show T1 weighted images of axial, coronal and sagittal slices, respectively. In patients who did not show deficit or recovered until postoperative 3 months, deep parts of the superior frontal gyrus and middle frontal gyrus were preserved except in Cases 5 and 16.
